# Adherence to hospital nutritional status monitoring and reporting guidelines

**DOI:** 10.1371/journal.pone.0204000

**Published:** 2018-09-21

**Authors:** Saman Khalatbari-Soltani, Pedro Marques-Vidal

**Affiliations:** 1 Institute of Social and Preventive Medicine (IUMSP), Lausanne, Switzerland; 2 Department of Medicine, Internal Medicine, Lausanne University Hospital (CHUV), Lausanne, Switzerland; Sefako Makgatho Health Sciences University, SOUTH AFRICA

## Abstract

**Aims of the study:**

Despite the widespread existence of guidelines regarding undernutrition monitoring and reporting, there is scarce information whether they are followed. We aimed to evaluate the adherence to guidelines regarding undernutrition monitoring and reporting as well as their determinants in a university hospital.

**Methods:**

Retrospective analysis of discharged patients with data on Nutritional Risk Screening score (NRS-2002) from the department of internal medicine of the Lausanne University Hospital for years 2013–14. Adherence to the hospital monitoring guidelines, i.e.: 1) discharged patients with NRS-2002 score≥3 should have prealbumin levels measured, and 2) discharged patients with prealbumin levels<0.20 g/l should be rechecked 7 days afterwards, was assessed. Reporting of nutritionally ‘at-risk’ status in the discharge letter was also assessed. Multivariable logistic regression was used to examine potential determinants of adherence to guidelines.

**Results:**

Of the 2,539 discharged patients with NRS-2002 data, 1,605 (63.0%) were nutritionally ‘at-risk’. Complete adherence to the monitoring guideline was observed in 238 (14.8%) of ‘at-risk’ patients. After multivariable analysis, adherence to the first step of monitoring guideline was associated with older age (≥ 80 years) [OR (95% CI): 2.03 (1.29–3.18)], high comorbidity index [1.36 (1.05–1.77)], and nutritional management [5.57 (4.38–7.07)]. Nutritional management was also associated with adherence to the second step of monitoring [3.98 (2.33–6.78)]. Adherence to the reporting guideline was observed in 343 (21.4%) of ‘at-risk’ patients. Multivariable analysis showed that adherence to the reporting guideline was associated with NRS-2002 score>4 [1.97 (1.47–2.64)], nutritional management [3.80 (2.85–5.07)], and adherence to the monitoring guideline [3.33 (2.35–4.71)].

**Conclusions:**

Our results show a poor adherence to guidelines regarding undernutrition monitoring and reporting, possibly due to lack of training, staff, and time.

## Introduction

Undernutrition is a common condition among hospitalized patients, varying between 20 and 60% depending on study population and definition applied [1,2]. Undernutrition or being ‘at-risk’ of undernutrition can deteriorate during hospitalization, impacting patients’ outcomes, quality of life, and health economics [3,4]. Thus, it is advisable that all patients admitted to hospital be screened, and that ‘at-risk’ and undernourished patients be adequately managed and monitored regularly [5,6]. Careful monitoring followed by proper nutrition intervention is vital for achievement of short- and long-term goals of nutrition care plan [7]. Still, several studies have shown that nutritional status is neither adequately screened, nor completely treated [1,4,5,8–10]. However, these studies mostly focused on screening and nutritional care rather than on monitoring. Finally, for economic and public health reasons, both the patients’ nutritional status and management should be accurately documented [6]. Moreover, proper documentation of undernutrition is a fundamental step for improving individualized care planning, disease monitoring, and healthcare costs estimation and reimbursement [11,12]. Of note, currently there is no single, universally accepted approach for undernutrition documentation in routine clinical practice [13]. Furthermore, several studies have shown that undernutrition or being nutritionally ‘at-risk’ is frequently not systematically documented [9,14–16].

Switzerland has one of the best health systems in the world [17], but the few data available suggest that screening for undernutrition is inadequately implemented [10,18]. Whether the same applies for monitoring and reporting has not been evaluated. Thus, we aimed to assess adherence to guidelines regarding undernutrition monitoring and reporting in a large university hospital. We also aimed to identify factors associated with adherence to these guidelines.

## Methods

### Study design and setting

This is a retrospective study using medical information from of the department of internal medicine of the Lausanne University Hospital (CHUV). The CHUV is one of the Swiss university hospitals (www.chuv.ch) and the Internal medicine unit of the CHUV is the largest in Switzerland, with over 4,000 admissions per year.

### Study sample

For the present analyses, we used hospitalized patients’ data from January 1^st^ 2013 to December 31^st^ 2014. This sampling period was chosen because the department started implementing a nutritional risk screening procedure using the Nutrition risk screening (NRS-2002) score in 2013. Data collection, merging, and coding was performed by the Lausanne university hospital team; investigators were blinded to patients’ identifiers.

All hospital discharges of adult patients (>18 years old) with NRS-2002 score data were considered as eligible. Of the 8,541 hospital discharges that occurred during the study period, 2,539 (29.7%) were considered as eligible, of whom 934 (36.8%) were excluded due to an NRS-2002 score <3 “**S1 Fig**”. Thus, the final sample consisted of 1,605 hospital discharges.

### Assessment of adherence to the hospital monitoring and reporting guidelines

The procedure regarding undernutrition screening and monitoring in the department of internal medicine is summarized in “**S2 Fig**”. The CHUV monitoring guidelines have a two-step approach: 1) all nutritionally ‘at risk’ patients should have their prealbumin levels measured, and 2) patients with prealbumin levels <0.20 g/l should have a second prealbumin measurement performed 7 days after the first one to monitor their nutritional status. Adherence to the CHUV monitoring guideline was assessed at those two steps; for step 2) the analysis period was extended to 10 days because of possible delays due to weekends or holidays.

The CHUV reporting guideline states that all nutritionally ‘at-risk’ patients should be reported as such in the discharge letter. This condition is then coded using the International Classification of Diseases 10^th^ revision (ICD-10). We systematically searched for all ICD-10 codes related to undernutrition and adherence to the reporting guideline was defined by presence of at least one undernutrition related codes “**S1 Table**”. If both criteria were not met, adherence to the reporting guideline were considered as not implemented.

### Nutrition risk screening and data collection procedure

Nutrition risk screening was defined by the presence of NRS-2002 scores in patients’ electronic medical record. The NRS-2002 is one of the most commonly used validated nutrition screening tools [1,19]. Briefly, it consists of the evaluation of the nutritional status (scored 0 to 3) and the disease severity (scored 0 to 3), with an extra score of 1 for patients older than 70 years. The scoring system is provided in “**S2 Table**”. The cores are added and scores ≥3 indicate that the patient is nutritionally ‘at-risk’. In this study, NRS-2002 scores were further categorized into medium (NRS-2002 score: 3–4) and high (NRS-2002 score >4) risk [20].

### Socio-demographic and clinical variables

We extracted the following explanatory variables from the patients discharge files: gender, age (categorized into three groups: 18–59, 60–79, and >80 years), and main diagnosis “**S3 Table**”. Severity of disease was assessed using the Swiss version of the Charlson Comorbidity Index (CCI) [21] and dichotomized into low (CCI<2) and high (CCI≥2) [22]. Height and weight were collected from the electronic records and used to calculate body mass index (BMI). Nutritional management was considered if the patient received enteral, parenteral, oral nutrition supplementation or specific dietary regimen.

### Statistical analysis

Statistical analyses were performed using Stata version 14.1 for Windows (Stata Corp, College Station, TX, USA). Statistical significance was assessed for a two-sided test with p-value <0.05. Descriptive results were expressed as average ± standard deviation for continuous data and as number of participants (percentage) for categorical data. Bivariate analysis was performed using logistic regression to identify potential determinants of adherence to guidelines. In this model, adherence to the guideline (yes or no) was the outcome and each socio- demographic or clinical characteristic was the predictor. Results were expressed as odds ratio (OR) and 95% confidence interval. Multivariable analysis was then performed using logistic regression to identify the factors significantly and independently associated with adherence to guidelines. Model fit for the multivariable analysis was tested using the Hosmer-Lemeshow goodness-of-fit test for each outcome. The p-value for the tests were not significant (>0.05), suggesting adequate model fit. The linear relationship with age with adherence to guidelines was evaluated categorically using orthogonal polynomial contrasts (command contrast p. of Stata) and continuously by calculating the odds ratio of a 10-year increase.

To investigate the potential effect of in-hospital mortality on adherence to the reporting guideline, analyses were performed after excluding patients who died during hospitalization. Further, in January 2014, the Swiss society of clinical nutrition issued a recommendation regarding undernutrition coding [23]. We thus adjusted for calendar year to take into account this change. Sensitivity analysis were conducted using discharged patients with a NRS-2002 ≥3 plus a BMI <18.5 kg/m^2^, which are the criteria suggested by the European Society of Parenteral and Enteral Nutrition (ESPEN) for definite undernutrition status [6].

### Ethics

The Ethics Committee of the Canton Vaud (www.cer-vd.ch, decision 428–14, of Dec. 2, 2014) and the CHUV board of directors (decision of Dec. 5, 2014) approved this study. Only routinely collected data was used. All information was extracted and anonymized by the hospital clinical research staff before being handled for analysis.

## Results

### Adherence to the monitoring guideline

Of the 1,605 nutritionally ‘at-risk’ cases (56.3% women; mean age 78.1 ± 14.3) “**S4 Table**”, 530 (33%) had their prealbumin levels measured. In bivariate analysis (**Table 1**), adherence to the first step of the monitoring guideline was positively associated with older age, having circulatory system disease, mental and behavioral disorder, NRS-2002 score>4, high CCI, and nutritional management. Except for circulatory system disease, the associations remained significant on multivariable analysis, which also showed a positive association with year of admission and a negative association with cancer and digestive system diseases.

**Table 1:** Factors associated with adherence to hospital guidelines regarding undernutrition monitoring (first step: Checking prealbumin level), department of internal medicine of the Lausanne university hospital, 2013 and 2014.

Characteristics	Bivariate analysis		Multivariate analysis	
	Unadjusted OR (95% CI)	P-value	Adjusted OR (95% CI)	P-value
**Admission year**				
2013	1 (ref.)		1 (ref.)	
2014	1.24 (0.98–1.58)	0.073	1.45 (1.11–1.88)	0.006
**Gender**				
Men	1 (ref.)		1 (ref.)	
Women	1.03 (0.83–1.27)	0.79	1.00 (0.79–1.26)	0.97
**Age category**				
18–59	1 (ref.)		1 (ref.)	
60–79	1.70 (1.12–2.60)	0.013	1.63 (1.03–2.58)	0.036
80+	2.05 (1.37–3.06)	<0.001	2.03 (1.29–3.18)	0.002
p-value for trend*	<0.001		0.002	
**Main diagnosis**				
Miscellaneous	1 (ref.)		1 (ref.)	
Circulatory system diseases	1.37 (1.01–1.87)	0.046	1.23 (0.86–1.76)	0.24
Cancer	0.82 (0.57–1.19)	0.30	0.51 (0.32–0.79)	0.003
Digestive system diseases	0.64 (0.39–1.02)	0.062	0.55 (0.33–0.92)	0.023
Infectious diseases	1.43 (0.94–2.19)	0.093	1.21 (0.76–1.93)	0.42
Mental & behavioral disorder/ Nervous system disease	1.64 (1.10–2.44)	0.015	1.74 (1.12–2.69)	0.013
Respiratory system diseases	1.31 (0.96–1.79)	0.092	1.24 (0.87–1.76)	0.22
**NRS-2002 categories**				
Medium (3–4)	1 (ref.)		1 (ref.)	
High (>4)	1.5 (1.17–1.91)	0.001	1.11 (0.84–1.46)	0.45
**Charlson comorbidity index**				
Low (CCI<2)	1 (ref.)		1 (ref.)	
High (CCI≥2)	1.30 (1.06–1.60)	0.013	1.36 (1.05–1.77)	0.020
**Any nutritional management**				
No	1 (ref.)		1 (ref.)	
Yes	4.95 (3.96–6.20)	<0.001	5.57 (4.38–7.07)	<0.001

Abbreviations: ref., reference category; CCI, Charlson comorbidity index; NRS-2002, nutrition risk screening 2002. Analyses were performed among patients with NRS-2002≥3 (n = 1,605). Bivariate analysis performed using logistic regression in which adherence to the guideline (yes or no) was the outcome and each characteristic was the predictor; multivariable analysis performed using logistic regression adjusting for all variables in the table.

* P-values for trend are computed using orthogonal polynomial contrasts (command contrast p. of Stata).

Among the 530 cases with prealbumin measurement, 439 (82.8%) had levels below 0.20 g/l. No associations were found between low prealbumin levels and their socio-demographic and clinical characteristics; similarly, no association was found between low prealbumin levels and nutritional management “**S5 Table**”.

Of the 439 cases with low prealbumin levels at the first measurement, 346 (78.8%) had a hospital stay ≥10 days and were thus eligible for a second measurement of prealbumin levels. Of the 346 eligible cases, 189 (54.6%) had a second prealbumin measurement. In bivariate analysis (**Table 2**), adherence to the second step of monitoring guideline was positively associated with having NRS-2002 score>4, high CCI, and nutritional management. In multivariable analysis (Table 2), only the association with nutritional management remained significant. Overall, full adherence to the hospital nutrition monitoring guideline (first and second steps) was found among only 209 (13.0%) of the 1,605 nutritionally ‘at-risk’ cases “**S3 Fig**”.

**Table 2:** Factors associated with adherence to hospital guidelines regarding undernutrition monitoring (second step: Re-checking prealbumin levels), department of internal medicine of the Lausanne university hospital, 2013 and 2014.

Characteristics	Bivariate analysis		Multivariable analysis	
	Unadjusted OR (95% CI)	P-value	Adjusted OR (95% CI)	P-value
**Admission year**				
2013	1 (ref.)		1 (ref.)	
2014	0.88 (0.54–1.44)	0.61	1.07 (0.63–1.84)	0.79
**Gender**				
Men	1 (ref.)		1 (ref.)	
Women	0.75 (0.49–1.15)	0.19	0.72 (0.45–1.16)	0.17
**Age category**				
18–59	1 (ref.)		1 (ref.)	
60–79	0.71 (0.26–1.91)	0.49	0.73 (0.24–2.23)	0.58
80+	0.60 (0.23–1.55)	0.29	0.73 (0.25–2.18)	0.57
p-value for trend*	0.24		0.31	
**Main diagnosis**				
Miscellaneous	1 (ref.)		1 (ref.)	
Circulatory system diseases	1.44 (0.77–2.69)	0.25	0.90 (0.43–1.89)	0.79
Cancer	1.92 (0.88–4.20)	0.10	0.99 (0.38–2.58)	0.98
Digestive system diseases	1.92 (0.69–5.35)	0.21	1.89 (0.58–6.13)	0.29
Infectious diseases	0.81 (0.35–1.86)	0.61	0.52 (0.21–1.27)	0.15
Mental & behavioral disorder/ Nervous system disease	2.43 (1.09–5.43)	0.03	1.88 (0.80–4.42)	0.14
Respiratory system diseases	1.28 (0.66–2.48)	0.46	0.94 (0.45–1.98)	0.87
**NRS-2002 categories**				
Medium (3–4)	1 (ref.)		1 (ref.)	
High (>4)	1.64 (1.02–2.65)	0.043	1.45 (0.85–2.48)	0.17
**Charlson comorbidity index**				
Low (CCI<2)	1 (ref.)		1 (ref.)	
High (CCI≥2)	1.71 (1.12–2.62)	0.014	1.73 (1.00–2.98)	0.050
**Any nutritional management**				
No	1 (ref.)		1 (ref.)	
Yes	3.90 (2.35–6.47)	<0.001	3.98 (2.33–6.78)	<0.001

Abbreviations: ref., reference category; CCI, Charlson comorbidity index; NRS-2002, nutrition risk screening 2002. Analyses were performed among patients with NRS-2002≥3, first-prealbumin levels<0.2 g/l and length of hospital stay ≥10 days (n = 346/439). Results are presented as OR (95% CI). Bivariate analysis performed using logistic regression in which adherence to the guideline (yes or no) was the outcome and each characteristic was the predictor; multivariable analysis performed using logistic regression adjusting for all variables in the table.

* P-values for trend are computed using orthogonal polynomial contrasts (command contrast p. of Stata).

### Adherence to reporting guideline

Among the 1,605 nutritionally ‘at-risk’ discharged patients, 343 (21.4%) had undernutrition-related ICD-10 codes in the discharge data “S3 Fig”. In bivariate analysis (**Table 3**), adherence to reporting guideline was positively associated with having infectious disease, NRS-2002 score>4, nutritional management, and adherence to the monitoring guideline. In multivariable analysis (Table 3), the associations remained significant except for infectious disease. Moreover, the multivariable analysis showed that adherence to reporting guideline was negatively associated with having cancer and diseases of the circulatory and respiratory system.

**Table 3:** Factors associated with undernutrition reporting in discharge data, department of internal medicine of the Lausanne university hospital, 2013 and 2014.

Characteristics	Bivariate analysis		Multivariable analysis	
	Unadjusted OR (95% CI)	P-value	Adjusted OR (95% CI)	P-value
**Admission year**				
2013	1 (ref.)		1 (ref.)	
2014	0.75 (0.58–0.98)	0.034	0.82 (0.61–1.1)	0.18
**Gender**				
Men	1 (ref.)		1 (ref.)	
Women	0.8 (0.63–1.01)	0.062	0.90 (0.68–1.18)	0.44
**Age category**				
18–59	1 (ref.)		1 (ref.)	
60–79	0.89 (0.59–1.34)	0.58	0.84 (0.53–1.32)	0.44
80+	0.65 (0.44–0.96)	0.030	0.67 (0.43–1.05)	0.08
p-value for trend*	0.005		0.08	
**Main Diagnosis**				
Miscellaneous	1 (ref.)		1 (ref.)	
Circulatory system diseases	0.39 (0.25–0.59)	<0.001	0.28 (0.17–0.45)	0.001
Cancer	1.01 (0.68–1.49)	0.96	0.56 (0.35–0.91)	0.020
Digestive system diseases	1.07 (0.67–1.71)	0.76	0.80 (0.48–1.33)	0.38
Infectious diseases	1.61 (1.04–2.5)	0.034	1.15 (0.70–1.88)	0.57
Mental & behavioral disorder/ Nervous system disease	0.91 (0.57–1.44)	0.67	0.68 (0.40–1.16)	0.15
Respiratory system diseases	0.79 (0.55–1.14)	0.21	0.57 (0.38–0.87)	<0.001
**NRS-2002 categories**				
Medium (3–4)	1 (ref.)		1 (ref.)	
High (>4)	2.51 (1.93–3.27)	<0.001	1.97 (1.47–2.64)	0.001
**Charlson comorbidity index**				
Low (CCI<2)	1 (ref.)		1 (ref.)	
High (CCI≥2)	1.11 (0.87–1.41)	0.40	1.10 (0.81–1.50)	0.53
**Any nutritional management**				
No	1 (ref.)		1 (ref.)	
Yes	5.37 (4.12–7.01)	<0.001	3.80 (2.85–5.07)	0.001
**Adherence to monitoring guideline**				
No	1 (ref.)		1 (ref.)	
Yes	4.91 (3.62–6.66)	<0.001	3.33 (2.35–4.71)	0.001

Abbreviations: ref., reference category; CCI, Charlson comorbidity index; NRS-2002, nutrition risk screening 2002. Analyses were performed among patients with NRS-2002≥3 (n = 1,605). Results are presented as OR (95% CI). Bivariate analysis performed using logistic regression in which adherence to the guideline (yes or no) was the outcome and each characteristic was the predictor; multivariable analysis performed using logistic regression adjusting for all variables in the table.

* P-values for trend are computed using orthogonal polynomial contrasts (command contrast p. of Stata).

Exclusion of those who died during hospitalization (n = 119) did not appreciably change the adherence to the reporting guideline (21.6%). Sensitivity analysis restricted to 193 patients with both NRS-2002 ≥3 and BMI <18.5 kg/m^2^ led to comparable results, although the associations of NRS-2002, cancer, and diseases of the respiratory system with undernutrition reporting were no longer significant “**S6 Table**”.

## Discussion

Less than one sixth of patients ‘at-risk’ of undernutrition was adequately monitored, and only one fifth was reported as such in the discharge letter. Our results indicate that adherence to guidelines regarding undernutrition monitoring and reporting is low in a Swiss hospital setting.

### Adherence to the monitoring guideline

Inadequate recognition and proper treatment of hospital undernutrition has prompted the issuing of guidelines for screening, management, monitoring, and documentation [10]. Still, there is no consensus regarding which biological markers should be used to diagnose and monitor undernutrition. The American Society for Parenteral and Enteral Nutrition mentions prealbumin and C-reactive protein as part of the ‘*nutrition programs in hospitals’* [24], and the recent ESPEN consensus statement indicates that albumin and prealbumin are indicators of undernutrition etiology [6]. Pre-albumin, together with albumin and C-reactive protein, is one of the general laboratory parameters used to assess patient’s food intake, appetite, nutrients absorption along with inflammatory activity itself [25]. Prealbumin is also a good marker to assess undernutrition prognosis and to evaluate the efficacy of nutritional management and refeeding [26].

The CHUV guidelines state that all nutritionally ‘at-risk’ patients (NRS-2002 score ≥3) should have their prealbumin levels measured. Still, our results indicate that this measurement is performed in only one third of them. Possible explanations for this low adherence rate are lack of manpower, excessive workload, inadequate nutrition knowledge, and no defined responsibilities [3,27], but no such data was collected in this study. Prealbumin was more frequently measured in patients of older age, diagnosed with mental and behavioral disorders, with a higher NRS-2002 score, and with a higher comorbidity index. The higher monitoring among older patients could be due to the large number of studies showing that nutritional interventions significantly reduce complications, length of hospital stay, hospital readmission and costs in patients aged >65 [7,28–30]. Also, a likely explanation for the higher monitoring among patients with mental and behavioral disorders is the high prevalence of swallowing disorders and more severe inflammation among these patients [25].

Adherence rate to the second step of the guidelines (i.e. re-checking prealbumin levels) was higher than to the first one. Nevertheless, it only concerned half of the patients, leading to a full adherence rate (i.e. to the first and the second steps) of only 13%. This value is lower than in Italy (21.6%) among 24 medical and surgical departments [31] or Cuba, where less than 20% of patients had their serum albumin levels assessed [32]. One questionnaire-based study among doctors and nurses in Denmark, Sweden and Norway also showed that less than one third of ‘at-risk’ patients was monitored regarding the effectiveness of nutritional management [16]. Overall, our results indicate that adherence to the monitoring guideline is not optimal. Improvements should be made to monitor (and manage) all nutritionally ‘at-risk’ patients as per the guidelines, which will likely require an increase in human resources. In the future, it will be of interest to evaluate the economic and health impact of hospital guidelines before they are issued.

### Adherence to reporting guideline

Only one out of five patients nutritionally ‘at-risk’ was reported as such in the discharge data. Our results agree with one study conducted in one university medical center in Amsterdam, where nutritional status was documented in only 15.5% of referral letters by the general practitioner [9]. Conversely, a study conducted by Meijers et al. showed that three out of four wards documented undernutrition and nutritional interventions in the medical records [14]. Possible explanations for the low reporting rate observed here include excessive workload or underrating of undernutrition status relative to other conditions by clinicians. Indeed, in many countries (including Switzerland), nurses and physicians have little training in nutrition, which is one of the major barriers regarding proper adherence to *‘nutrition programs in hospital’* [5,18,33,34].

Cancer patients who were nutritionally ‘at-risk’ had a lower likelihood of being reported as such. This finding is in agreement with a previous study which reported that undernutrition is underestimated among cancer patients [35] or to the fact that the impact of nutrition intervention on cancer therapy is frequently underestimated [36].

In January 2014, the Swiss society of clinical nutrition issued several recommendations regarding undernutrition reporting [23]. Still, no improvement was found between 2013 and 2014 regarding the reporting rates of nutritionally ‘at-risk’ patients. Our results suggest that either the recommendation was not implemented, or it took longer than one year to be implemented.

Overall, our results highlight that adherence to the reporting guideline is inadequate.

### Impact and solutions

Monitoring patient’s nutritional status is imperative to check the effectiveness of the nutritional care plans and medical treatments, especially among patients who are ‘at-risk’ of undernutrition [1,37]. Undernutrition monitoring improves quality of care, patient’s clinical outcomes and reduces length of hospital stay and hospital re-admission [7]. Moreover, accurate documentation of diagnoses and medical procedures is fundamental to improve individualized care planning [11,12], disease monitoring, and healthcare costs estimation and reimbursement [3,38]. Possible solutions to better reporting include stronger implementation of local guidelines, provision of training to health care professionals [5,18], and automatic system to track both monitoring [12], and reporting of patient’s nutritional status in the discharge letter.

### Strengths and limitations

The strength of this study is its large sample size of ‘at-risk’ or undernourished patients. The main limitations are that it was restricted to a single department of one university hospital in Switzerland; as there is no consensus regarding undernutrition monitoring guidelines, generalizability is thus limited. Still, our results provide the first evaluation of adherence to guidelines on monitoring and reporting of undernutrition status, and its methodology could be replicated in other health care settings. Second, it is possible that the hospital staff unconsciously selected the most nutritionally ‘at-risk’ patients for screening, thus leading to a higher prevalence of the condition. Still, we were interested in the implementation of the monitoring and the reporting guidelines rather than the screening itself. Hence, even in the presence of a selection bias, our results indicate that adherence to the guidelines regarding nutrition monitoring and reporting is far from optimal.

## Conclusion

In a Swiss hospital setting, less than one sixth of patients ‘at-risk’ of undernutrition was adequately monitored, and only one fifth was reported as such in the discharge letter. Adherence to guidelines regarding undernutrition monitoring and reporting varies according to the patients’ characteristics and could be due to lack of training, staff, and time. Implementation measures are urgently required to improve management of undernourished patients and to estimate prevalence of undernutrition using hospital discharge data.

## Supporting information

S1 Fig Participant selection procedure. Abbreviations: NRS-2002, nutrition risk screening.
(TIF)

S2 Fig Undernutrition monitoring and reporting guideline at the department of internal medicine of the Lausanne university hospital. Abbreviations: NRS-2002, nutrition risk screening 2002; ICD-10, International Classification of Diseases, 10^th^ revision; LOS, length of hospital stay.
(TIF)

S3 Fig Adherence to the undernutrition monitoring and reporting guideline at department of internal medicine of the Lausanne university hospital. Abbreviations: NRS-2002, nutrition risk screening 2002; ICD-10, International Classification of Diseases, 10^th^ revision.
(TIF)

S1 Table International Classification of Diseases, 10^th^ revision codes used to define undernutrition, department of internal medicine of the Lausanne university hospital, 2013 and 2014. Abbreviation: ICD-10, International Classification of Diseases 10th revision.
(DOCX)

S2 Table Nutrition risk screening scoring procedure. Adopted from Kondrup et al., 2002, doi: 10.1177/0884533617692527.
(DOCX)

S3 Table International Classification of Diseases, 10^th^ revision codes used to categorize the main diagnosis at discharge, department of internal medicine of the Lausanne university hospital, 2013 and 2014. Abbreviation: ICD-10, International Classification of Diseases 10th revision.
(DOCX)

S4 Table Socio-demographic and clinical characteristics of included and excluded patients, department of internal medicine of the Lausanne university hospital, 2013 and 2014. Abbreviation: CCI, Charlson comorbidity index. Analyses were performed among patients with NRS-2002 score in their medical file (n = 2,539). Results are expressed as number of participants (column %) except for ^a^ where prevalence is expressed as number of patients (row %) or as average ± standard deviation. P-value for comparisons between groups performed using chi-square for categorical variables and student’s t-test for continuous variables.
(DOCX)

S5 Table Bivariate and multivariate analysis of the factors associated with low prealbumin levels (first measurement), department of internal medicine of the Lausanne university hospital, 2013 and 2014. Abbreviations: ref., reference category; CCI, Charlson comorbidity index; NRS-2002, nutrition risk screening 2002. Analyses were performed among patients with NRS-2002≥3 and first prealbumin measurement (n = 530). Results for bivariate analysis are presented as number of patients (column %) and for multivariate analysis as OR (95% CI). Bivariate analysis performed using chi-square or Fisher’s exact test (ⱡ); multivariable analysis performed using logistic regression adjusting for all variables in the table.
(DOCX)

S6 Table Factors associated with undernutrition reporting, restricting the analysis to patients with a body mass index <18.5 kg/m^2^, department of internal medicine of the Lausanne university hospital, 2013 and 2014. Abbreviations: ref., reference category; CCI, Charlson comorbidity index; NRS-2002, nutrition risk screening 2002. Analyses were performed among patients with NRS-2002≥3 and body mass index<18.5 kg/m^2^ (n = 193). Results are presented as OR (95% CI). Bivariate analysis performed using logistic regression analysis in which adherence to the guideline (yes or no) was an outcome and each characteristic was a predictor; multivariable analysis performed using logistic regression adjusting for all variables in the table. * P-values for trend are presented, for which an ordinal variable was included as a continuous term in a logistic regression model.
(DOCX)

S1 File STROBE checklist-checklist of items that should be included in reports of cross-sectional studies. * Give information separately for exposed and unexposed groups. Note: An Explanation and Elaboration article discusses each checklist item and gives methodological background and published examples of transparent reporting. The STROBE checklist is best used in conjunction with this article (freely available on the Web sites of PLoS Medicine at http://www.plosmedicine.org/, Annals of Internal Medicine at http://www.annals.org/, and Epidemiology at http://www.epidem.com/). Information on the STROBE Initiative is available at www.strobe-statement.org.
(DOC)
