# Supplementary figures and images for: Adherence to hospital nutritional status monitoring and reporting guidelines
Source: PLoS One. 2018 Sep 21;13(9):e0204000. doi: 10.1371/journal.pone.0204000 (PMC6150473; doi:10.1371/journal.pone.0204000)

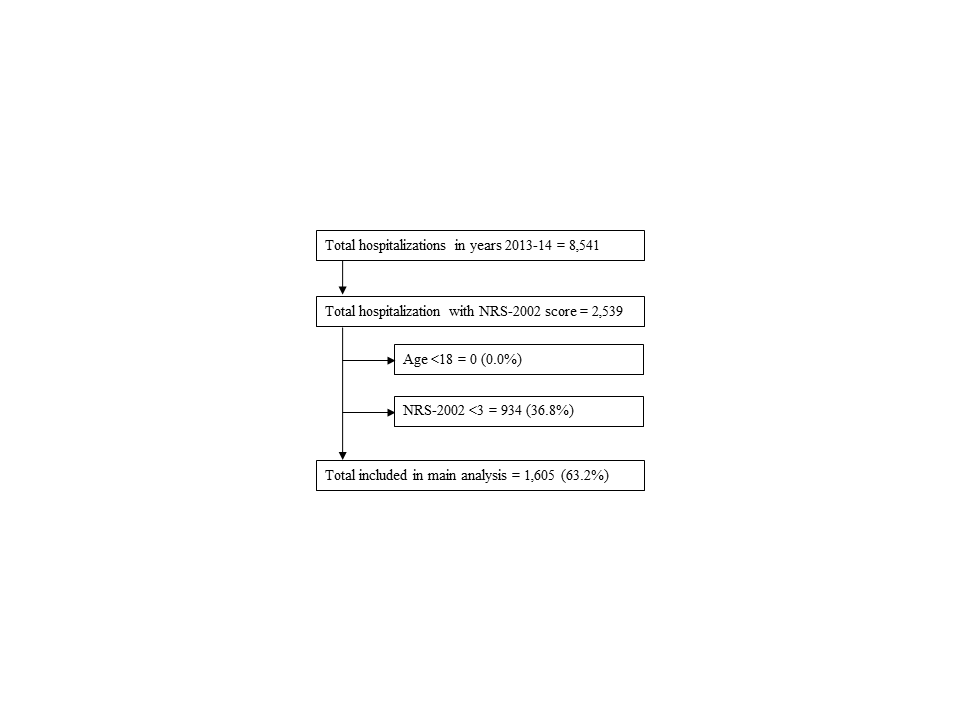

Supplement: S1 Fig — Abbreviations: NRS-2002, nutrition risk screening. (TIF) [file pone.0204000.s001.tif]

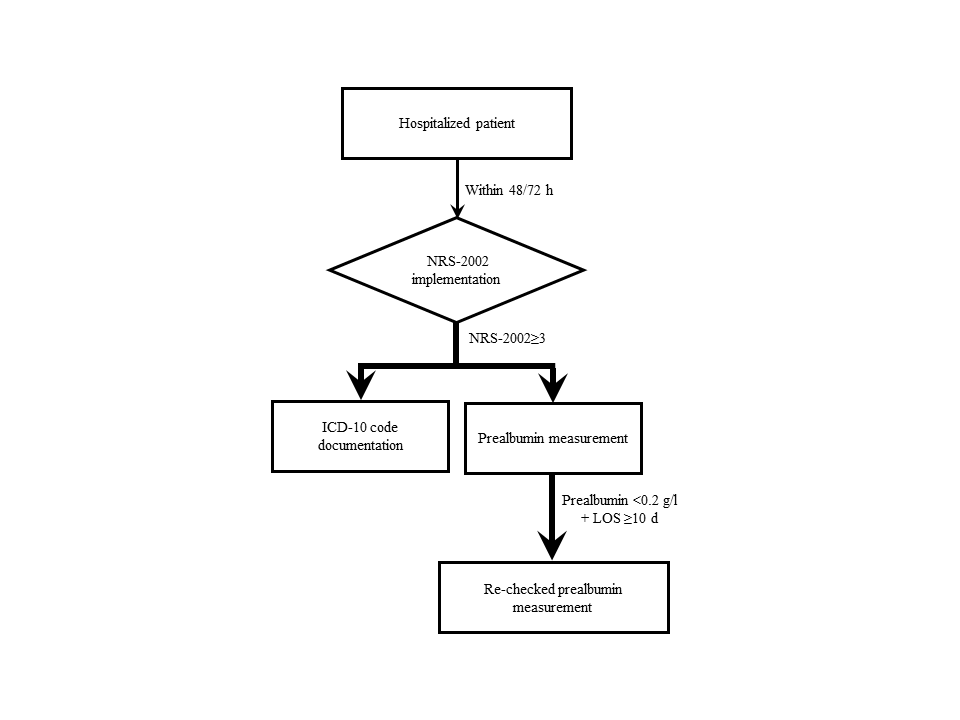

Supplement: S2 Fig — Abbreviations: NRS-2002, nutrition risk screening 2002; ICD-10, International Classification of Diseases, 10th revision; LOS, length of hospital stay. (TIF) [file pone.0204000.s002.tif]

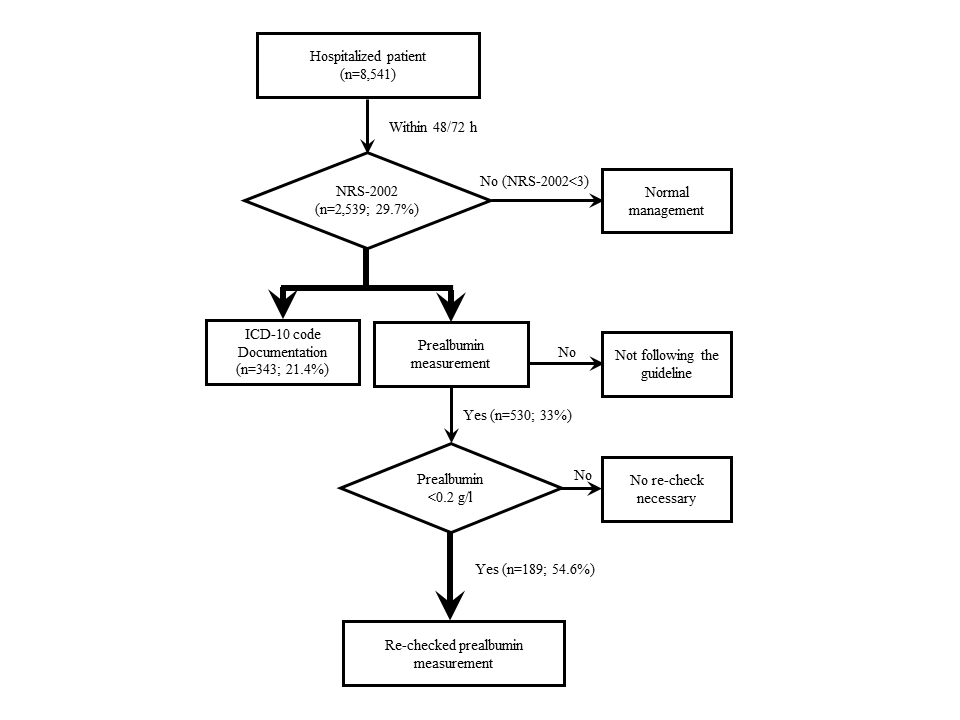

Supplement: S3 Fig — Abbreviations: NRS-2002, nutrition risk screening 2002; ICD-10, International Classification of Diseases, 10th revision. (TIF) [file pone.0204000.s003.tif]
