# Supplementary material for: Adherence to hospital nutritional status monitoring and reporting guidelines
Source: PLoS One. 2018 Sep 21;13(9):e0204000. doi: 10.1371/journal.pone.0204000 (PMC6150473; doi:10.1371/journal.pone.0204000)
Supplement: S1 Table — Abbreviation: ICD-10, International Classification of Diseases 10th revision. (DOCX) [file pone.0204000.s004.docx]

**S1Table. International Classification of Diseases, 10^th^ revision codes used to define undernutrition, department of internal medicine of the Lausanne university hospital, 2013 and 2014.**

| **ICD-10 code** | **Definition** |
| --- | --- |
| E40 | Kwashiorkor |
| E41 | Nutritional marasmus |
| E42 | Marasmic kwashiorkor |
| E43 | Unspecified severe protein-energy malnutrition |
| E44 | Protein-energy malnutrition of moderate and mild degree |
| E45 | Retarded development following protein-energy malnutrition |
| E46 | Unspecified protein-energy malnutrition |
| R63 | Symptoms and signs concerning food and fluid intake |
| R64 | Cachexia |

Abbreviation: ICD-10, International Classification of Diseases 10^th^ revision.
