# Supplementary material for: Adherence to hospital nutritional status monitoring and reporting guidelines
Source: PLoS One. 2018 Sep 21;13(9):e0204000. doi: 10.1371/journal.pone.0204000 (PMC6150473; doi:10.1371/journal.pone.0204000)
Supplement: S2 Table — Adopted from Kondrup et al., 2002, doi: 10.1177/0884533617692527. (DOCX) [file pone.0204000.s005.docx]

**S2 Table. Nutrition Risk screening scoring procedure**

|  |  | **Score** |
| --- | --- | --- |
| **Impaired nutritional status** | |  |
| Absent | Normal nutritional status | 0 |
| Mild | Weight loss >5% in 3 months OR  Food intake below 50–75% of normal requirement in preceding week | 1 |
| Moderate | Weight loss >5% in 2 months OR  BMI 18.5–20.5 + impaired general condition OR  Food intake 25–60% of normal requirement in preceding week | 2 |
| Severe | Weight loss >5% in 1 month (>15% in 3 months) OR  BMI <18.5 + impaired general condition OR  Food intake 0-25% of normal requirement in preceding week | 3 |
| **Severity of disease** | |  |
| Absent | Normal nutritional requirements | 0 |
| Mild | Hip fracture; chronic patients, in particular with acute complications: cirrhosis; chronic obstructive pulmonary disease; chronic hemodialysis; diabetes; cancer | 1 |
| Moderate | Major abdominal surgery; stroke; severe pneumonia; leukemia | 2 |
| Severe | Head injury; bone marrow transplantation; intensive care patients (APACHE>10). | 3 |
|  | Total Score |  |
| **Age** | If ≥70 years: add 1 to total score above | 1 |
|  | Age-adjusted total score |  |

Adopted from Kondrup et al., 2002, doi: 10.1177/0884533617692527
