# Supplementary material for: Adherence to hospital nutritional status monitoring and reporting guidelines
Source: PLoS One. 2018 Sep 21;13(9):e0204000. doi: 10.1371/journal.pone.0204000 (PMC6150473; doi:10.1371/journal.pone.0204000)
Supplement: S3 Table — Abbreviation: ICD-10, International Classification of Diseases 10th revision. (DOCX) [file pone.0204000.s006.docx]

**S3 Table. International Classification of Diseases, 10^th^ revision codes used to categorize the main diagnosis at discharge, department of internal medicine of the Lausanne university hospital, 2013 and 2014.**

| **ICD-10 codes** | **Main diagnosis** |
| --- | --- |
| A00-B00 | Infectious diseases |
| C00-D09 | Cancer |
| F00-F99; G00-G99 | Mental & behavioral disorder/ Nervous system disease |
| I00-I99 | Circulatory system diseases |
| J00-J99 | Respiratory system diseases |
| K00-K93 | Digestive system diseases |
| All others | Miscellaneous |

Abbreviation: ICD-10, International Classification of Diseases 10^th^ revision.
