# Supplementary material for: Adherence to hospital nutritional status monitoring and reporting guidelines
Source: PLoS One. 2018 Sep 21;13(9):e0204000. doi: 10.1371/journal.pone.0204000 (PMC6150473; doi:10.1371/journal.pone.0204000)
Supplement: S4 Table — Abbreviation: CCI, Charlson comorbidity index. Analyses were performed among patients with NRS-2002 score in their medical file (n = 2,539). Results are expressed as number of participants (column %) except for a where prevalence is expressed as number of patients (row %) or as average ± standard deviation. P-value for comparisons between groups performed using chi-square for categorical variables and student’s t-test for continuous variables. (DOCX) [file pone.0204000.s007.docx]

**S4 Table. Socio-demographic and clinical characteristics of included and excluded patients, department of internal medicine of the Lausanne university hospital, 2013 and 2014.**

| Characteristics | **Included**  (n=1,605) | **Excluded**  (n=934) | **P-value** |
| --- | --- | --- | --- |
| **Admission year** |  |  | 0.37 |
| 2013 | 433 (27.0) | 237 (25.4) |  |
| 2014 | 1172 (73.0) | 697 (74.6) |  |
| **Gender** |  |  | <0.001 |
| Men | 701 (43.7) | 491 (52.6) |  |
| Women | 904 (56.3) | 443 (47.4) |  |
| **Age** | 78.1 ± 14.3 | 71.4 ±15.6 | 0.001 |
| **Age category** |  |  | <0.001 |
| 18-59 | 159 (9.9) | 217 (23.2) |  |
| 60-79 | 515 (32.1) | 368 (39.4) |  |
| 80+ | 931 (58.0) | 349 (37.4) |  |
| **Main diagnosis** |  |  | 0.001 |
| Miscellaneous | 468 (29.2) | 305 (32.7) |  |
| Circulatory system diseases | 289 (18.0) | 210 (22.5) |  |
| Cancer | 201 (12.5) | 73 (7.8) |  |
| Digestive system diseases | 120 (7.5) | 66 (7.1) |  |
| Infectious diseases | 117 (7.3) | 67 (7.2) |  |
| Mental & behavioral disorder/ Nervous system disease | 132 (8.2) | 72 (7.7) |  |
| Respiratory system diseases | 278 (17.3) | 141 (15.1) |  |
| **Charlson comorbidity index categories** |  |  | <0.001 |
| Low (CCI<2) | 837 (52.1) | 562 (60.2) |  |
| High (CCI≥2) | 768 (47.9) | 372 (39.8) |  |

Abbreviation: CCI, Charlson comorbidity index. Analyses were performed among patients with NRS-2002 score in their medical file (n=2,539). Results are expressed as number of participants (column %) or as average ± standard deviation. P-value for comparisons between groups performed using chi-square for categorical variables and student’s t-test for continuous variables.
