# Supplementary material for: Adherence to hospital nutritional status monitoring and reporting guidelines
Source: PLoS One. 2018 Sep 21;13(9):e0204000. doi: 10.1371/journal.pone.0204000 (PMC6150473; doi:10.1371/journal.pone.0204000)
Supplement: S5 Table — Abbreviations: ref., reference category; CCI, Charlson comorbidity index; NRS-2002, nutrition risk screening 2002. Analyses were performed among patients with NRS-2002≥3 and first prealbumin measurement (n = 530). Results for bivariate analysis are presented as number of patients (column %) and for multivariate analysis as OR (95% CI). Bivariate analysis performed using chi-square or Fisher’s exact test (ⱡ); multivariable analysis performed using logistic regression adjusting for all variables in the table. (DOCX) [file pone.0204000.s008.docx]

**S5 Table. Bivariate and multivariate analysis of the factors associated with low prealbumin levels (first measurement), department of internal medicine of the Lausanne university hospital, 2013 and 2014.**

| Characteristics | **First prealbumin level** | | **P-value** | **Multivariable analysis** | **P-value** |
| --- | --- | --- | --- | --- | --- |
|  | **Normal**  **(≥0.2 g/l)** | **Low**  **(<0.2 g/l)** |  |  |  |
|  | (n=91) | (n=439) |  |  |  |
| **Admission year** |  |  | 0.032 |  |  |
| 2013 | 14 (15.4) | 114 (26.0) |  | 1 (ref.) |  |
| 2014 | 77 (84.6) | 325 (74.0) |  | 0.56 (0.30 - 1.05) | 0.069 |
| **Gender** |  |  | 0.31 |  |  |
| Men | 35 (38.5) | 194 (44.2) |  | 1 (ref.) |  |
| Women | 56 (61.5) | 245 (55.8) |  | 0.82 (0.51 - 1.34) | 0.43 |
| **Age category** |  |  | 0.64 ^ⱡ^ |  |  |
| 18-59 | 7 (7.7) | 27 (6.2) |  | 1 (ref.) |  |
| 60-79 | 25 (27.5) | 138 (31.4) |  | 1.53 (0.58 - 4.03) | 0.39 |
| 80+ | 59 (64.8) | 274 (62.4) |  | 1.53 (0.59 - 3.96) | 0.38 |
| p-value for trend |  |  |  | 0.38 |  |
| **Main Diagnosis** |  |  | 0.81 ^ⱡ^ |  |  |
| Miscellaneous | 30 (33) | 112 (25.5) |  | 1 (ref.) |  |
| Circulatory system diseases | 20 (22) | 88 (20.1) |  | 1.27 (0.62 - 2.58) | 0.50 |
| Cancer | 8 (8.8) | 45 (10.3) |  | 1.80 (0.67 - 4.85) | 0.24 |
| Digestive system diseases | 3 (3.3) | 23 (5.2) |  | 2.14 (0.57 - 8.09) | 0.26 |
| Infectious diseases | 7 (7.7) | 38 (8.7) |  | 1.35 (0.53 - 3.49) | 0.53 |
| Mental & behavioral disorder/ Nervous system disease | 9 (9.9) | 46 (10.5) |  | 1.25 (0.54 - 2.89) | 0.59 |
| Respiratory system diseases | 14 (15.4) | 87 (19.8) |  | 1.67 (0.80 - 3.51) | 0.17 |
| **NRS-2002 categories** |  |  | 0.50 |  |  |
| Medium (NRS=3-4) | 69 (75.8) | 318 (72.4) |  | 1 (ref.) |  |
| High (NRS>4) | 22 (24.2) | 121 (27.6) |  | 1.00 (0.58 - 1.73) | 0.98 |
| **Charlson comorbidity index** |  |  | 0.91 |  |  |
| Low (CCI<2) | 43 (47.3) | 210 (47.8) |  | 1 (ref.) |  |
| High (CCI≥2) | 48 (52.8) | 229 (52.2) |  | 0.78 (0.45 - 1.35) | 0.37 |
| **Any nutritional management** |  |  | 0.11 |  |  |
| No | 36 (39.6) | 136 (31.0) |  | 1 (ref.) |  |
| Yes | 55 (60.4) | 303 (69.0) |  | 1.38 (0.85 - 2.24) | 0.18 |

Abbreviations: ref., reference category; CCI, Charlson comorbidity index; NRS-2002, nutrition risk screening 2002. Analyses were performed among patients with NRS-2002≥3 and first prealbumin measurement (n=530). Results for bivariate analysis are presented as number of patients (column %) and for multivariate analysis as OR (95% CI). Bivariate analysis performed using chi-square or Fisher’s exact test (ⱡ); multivariable analysis performed using logistic regression adjusting for all variables in the table.
