# Supplementary material for: Adherence to hospital nutritional status monitoring and reporting guidelines
Source: PLoS One. 2018 Sep 21;13(9):e0204000. doi: 10.1371/journal.pone.0204000 (PMC6150473; doi:10.1371/journal.pone.0204000)
Supplement: S6 Table — Abbreviations: ref., reference category; CCI, Charlson comorbidity index; NRS-2002, nutrition risk screening 2002. Analyses were performed among patients with NRS-2002≥3 and body mass index<18.5 kg/m2 (n = 193). Results are presented as OR (95% CI). Bivariate analysis performed using logistic regression analysis in which adherence to the guideline (yes or no) was an outcome and each characteristic was a predictor; multivariable analysis performed using logistic regression adjusting for all variables in the table. * P-values for trend are presented, for which an ordinal variable was included as a continuous term in a logistic regression model. (DOCX) [file pone.0204000.s009.docx]

**S6 Table. Factors associated with undernutrition reporting, restricting the analysis to patients with a body mass index <18.5 kg/m^2^, department of internal medicine of the Lausanne university hospital, 2013 and 2014.**

| Characteristics | **Bivariate analysis** | | **Multivariable analysis** | |
| --- | --- | --- | --- | --- |
|  | **Unadjusted OR**  **(95% CI)** | **P-value** | **Adjusted OR**  **(95% CI)** | **P-value** |
| **Admission year** |  |  |  |  |
| 2013 | 1 (ref.) |  | 1 (ref.) |  |
| 2014 | 0.62 (0.31 - 1.24) | 0.17 | 0.55 (0.24 - 1.27) | 0.16 |
| **Gender** |  |  |  |  |
| Men | 1 (ref.) |  | 1 (ref.) |  |
| Women | 0.46 (0.25 - 0.85) | 0.013 | 0.58 (0.27 - 1.23) | 0.15 |
| **Age category** |  |  |  |  |
| 18-59 | 1 (ref.) |  | 1 (ref.) |  |
| 60-79 | 1.07 (0.45 - 2.56) | 0.88 | 0.90 (0.33 - 2.44) | 0.82 |
| 80+ | 0.67 (0.30 - 1.48) | 0.31 | 0.58 (0.21 - 1.55) | 0.27 |
| p-value for trend* | 0.18 |  | 0.15 |  |
| **Main Diagnosis** |  |  |  |  |
| Miscellaneous | 1 (ref.) |  | 1 (ref.) |  |
| Circulatory system diseases | 0.27 (0.09 - 0.82) | 0.021 | 0.19 (0.05 - 0.72) | 0.014 |
| Cancer | 1.35 (0.51 - 3.52) | 0.54 | 0.95 (0.27 - 3.34) | 0.94 |
| Digestive system diseases | 0.54 (0.16 - 1.82) | 0.32 | 0.38 (0.09 - 1.59) | 0.18 |
| Infectious diseases | 6.92 (0.82 - 58.34) | 0.075 | 5.97 (0.64 - 55.8) | 0.11 |
| Mental & behavioral disorder/ Nervous system disease | 0.95 (0.36 - 2.53) | 0.92 | 0.42 (0.13 - 1.42) | 0.16 |
| Respiratory system diseases | 0.91 (0.41 - 2.03) | 0.82 | 0.77 (0.31 - 1.89) | 0.56 |
| **NRS-2002 categories** |  |  |  |  |
| Medium (3-4) | 1 (ref.) |  | 1 (ref.) |  |
| High (>4) | 1.48 (0.83 - 2.64) | 0.18 | 1.43 (0.71 - 2.89) | 0.31 |
| **Charlson comorbidity index** |  |  |  |  |
| Low (CCI<2) | 1 (ref.) |  | 1 (ref.) |  |
| High (CCI≥2) | 1.13 (0.64 - 1.99) | 0.68 | 0.90 (0.41 - 1.94) | 0.78 |
| **Any nutritional management** |  |  |  | - |
| No | 1 (ref.) |  | 1 (ref.) |  |
| Yes | 3.16 (1.74 - 5.76) | <0.001 | 2.55 (1.26 - 5.15) | 0.009 |
| **Adherence to monitoring guideline** |  |  |  |  |
| No | 1 (ref.) |  | 1 (ref.) |  |
| Yes | 4.31 (1.92 - 9.66) | <0.001 | 4.67 (1.74 - 12.5) | 0.002 |

Abbreviations: ref., reference category; CCI, Charlson comorbidity index; NRS-2002, nutrition risk screening 2002. Analyses were performed among patients with NRS-2002≥3 and body mass index<18.5 kg/m^2^ (n=193). Results are presented as OR (95% CI). Bivariate analysis performed using logistic regression analysis in which adherence to the guideline (yes or no) was an outcome and each characteristic was a predictor; multivariable analysis performed using logistic regression adjusting for all variables in the table. * P-values for trend are computed using orthogonal polynomial contrasts (command contrast p. of Stata).
